# Optical coherence tomography: an assessment of current training across all levels of seniority in 8 ophthalmic units in the united kingdom

**DOI:** 10.1186/1471-2415-6-33

**Published:** 2006-10-24

**Authors:** Wai H Chan, John S Shilling, Michel Michaelides

**Affiliations:** 1 Department of Ophthalmology, St Thomas' Hospital, London, SE1 7EH, UK; 2 Moorfields Eye Hospital, City Road, London, EC1V 2PD, UK

## Abstract

**Background:**

Optical Coherence Tomography (OCT) is becoming an increasingly integral part of ophthalmological clinical practice. The accurate interpretation of OCT images is important both in terms of diagnosis and in directing subsequent management. The aim of this study was to determine the clinical competence in OCT image interpretation of ophthalmologists in different subspecialties and grades.

**Methods:**

Eight OCT images demonstrating a single macular pathology and two normal scans were selected by case notes review. These ten images were shown to thirty doctors and 10 non-medical staff from eight units. They were asked to identify each lesion, the average thickness of the lesion, and the axis at which the OCT was taken. One point was awarded for each correct answer.

**Results:**

The mean scores for the correct qualitative identification of the OCT lesion (with a maximum score of 10) for different grades of doctors and non-medical staff were as follows: medical retinal consultants (MRC), 9 (range, 8–10); vitreoretinal consultants (VRC), 7 (range, 6–9); non-retinal consultants (NRC), 4 (range, 2–6); vitreoretinal fellows (VRF), 4 (range, 3–7); specialist registrars (SpR), 3 (range, 2–5); senior house officers (SHO), 4 (range, 3–6); orthoptists, 1 (range, 0–1); ancillary staff, 2 (range, 0–3).

**Conclusion:**

A wide range in the ability to accurately interpret OCT images has been demonstrated. All doctors would thereby benefit from further training in the interpretation of OCT scans.

## Background

Humphrey optical coherence tomography (OCT) (Carl Zeiss Meditec, Inc. California) uses low-coherence interferometry to produce two-dimensional images of optical scattering from ocular tissues. OCT can detect reflected infrared light signals as small as approximately 10^-10 ^of the incident optical power [1]. The magnitude of resolution and penetration depth allow discrimination of discrete retinal layers, including the nerve fibre layer, photoreceptors, retinal pigment epithelium, Bruchs membrane and the choroid [2]. The high definition of OCT also makes it a valuable research tool in examining the biophysical profiles and elastic properties of Bruchs membrane [3].

Optical coherence tomography is now commonly being incorporated into medical and surgical retinal assessments of ophthalmological conditions. The high-resolution optical cross sectional images generated are increasingly being used to aid diagnosis and subsequent management in a wide range of retinal pathology, including central serous chorioretinopathy, retinal pigment epithelial detachments, cystoid macular oedema, epiretinal membranes and clinically significant macula oedema [1,4,5]. OCT scans provide an objective qualitative and quantitative retinal assessment which is clearly advantageous in the effective management of patients, both in terms of diagnosis and monitoring of response to therapy, especially when compared to the wide inter- and intra-observer variation in subjective assessments performed by clinicians.

OCT scans are increasingly being carried out by nursing, photographic and technical staff, with subsequent interpretation by the requesting clinician. Due to the increasing importance of OCT in ophthalmological practice we have undertaken an investigational study to assess the current standard of OCT scan interpretation, including the ability to correctly diagnose retinal pathology, determine the axis of the scan and estimate retinal thickness.

## Methods

**Figure 1:**
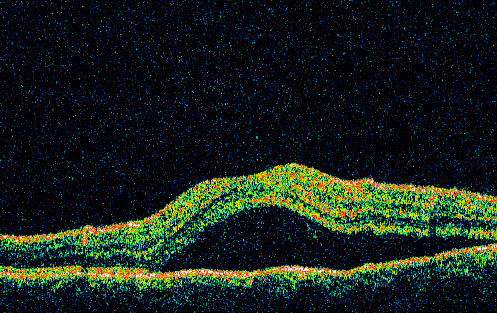
OCT image demonstrating central serous chorioretinopathy in a left eye, taken through the 180 degree axis.

Eight patients who had undergone OCT imaging for a single macular pathology in their left eye were selected by case notes review. The retinal disorders chosen were central serous chorioretinopathy (CSR) (Figure 1), pigment epithelial detachment (PED), subretinal neovascular membrane (SRNVM), cystoid macular oedema (CMO), posterior vitreous detachment (PVD), full thickness macula hole (FTMH), epiretinal membrane (ERM) and rhegmatogenous retinal detachment involving the macula (RRD). These diagnoses were made by a consultant ophthalmologist with a specialist retinal interest (JSS); who also had access to clinical data and where relevant, fundus fluorescein angiography findings. Patients with dual pathologies were excluded. Two scans without retinal pathology (Normal, N) were also included as controls, making a total of 10 OCT images. The OCT images shown to the clinicians did not include the retinal thickness or axis measurements that are provided by the software.

OCT was used to record a single fast macular thickness map. The scan length was limited to 6.0 mm in diameter. All scans were taken through the fovea along the 180-degree axis with the patient seated at the optimal height. Retinal map analysis support 4.0.1 software was used for the OCT report. Maximal thickness measurements were made at the centre of the fovea from retinal pigment epithelium to the inner limiting membrane. The maximal thickness of each scan used in the study, within the central 1.0 mm area, was: CSR = 295 um, PED = 300 um, SRNVM = 353 um, CMO = 407 um, PVD = 180 um, FTMH = 0 um, ERM = 250 um, RRD = 718 um, Normal = 190 um. Scans with signal strength <6 (max 10) were excluded. The average of 5 scans was used for each patient's parameters.

A total of 30 doctors were questioned, consisting of 5 medical retinal consultants (MRC), 5 vitreoretinal consultants (VRC), 5 non-retinal consultants (NRC), 5 vitreoretinal fellows (VRF), 5 specialist registrars (SpR) and 5 senior house officers (SHO). SpRs and SHOs were further subdivided into their respective years of experience (range Y1 to Y4). In addition, since non-medical staff are increasingly performing the OCT scans, 5 orthoptists and 5 ancillary staff (consisting of 4 nurses and one health care assistant) also participated in the study. Each participant was shown the ten OCT scans and asked for a diagnosis, the axis of the scan, and to estimate the maximum thickness, for each image; with one point awarded for each correct answer. Only one possible answer was accepted for the diagnosis, and an axis within 10 degrees and a thickness within 50 microns were deemed correct. Each subject was given a maximum of 1 minute to interpret each scan. Subjects were recruited from 8 centres across the UK including, St Thomas' Hospital, Queen Mary's Hospital, Whipps Cross Hospital, Moorfields Eye Hospital, Bradford Royal Infirmary, Cheltenham General Hospital, Kings College Hospital and The Western Eye Hospital. The protocol of the study was approved by the local Ethics Committee.

## Results

### Results for the correct OCT diagnoses

**Figure 2:**
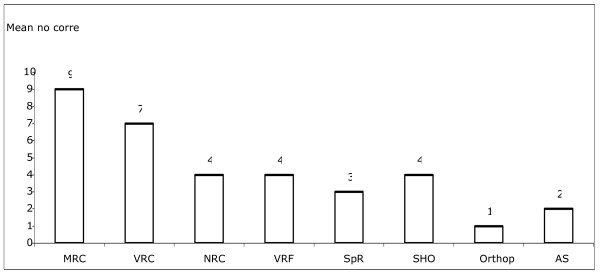
Mean scores for correct diagnosis out of 10 images for different grades of doctors.

The mean scores for correct diagnosis out of 10 images for different grades of doctors and non-medical staff were as follows: MRC 9 (range, 8–10); VRC 7 (range, 6–9); NRC 4 (range, 2–6); VRF 4 (range, 3–7); SpR 3 (range, 2–5); SHO 4 (range, 3–6); orthoptists 1 (range, 0–1); ancillary staff 2 (range, 0–3) (Figure 2).

### Results for the thickness of scans

**Figure 3:**
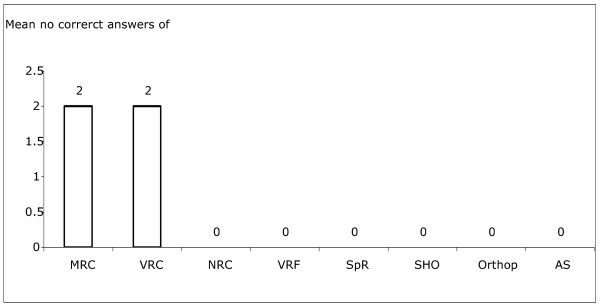
Mean scores for correct thickness measurements out of 10 images for different grades of doctors.

Two VRCs and one Y3 SHO completed this section of the survey. The two VRCs were able to estimate the average thickness of the normal scan to within 20 um. All other answers were more than 50 um from the correct thickness. All answers from the Y3 SHO were either more or less than 50 um from the correct thickness. The remaining participants were unable to estimate a thickness (Figure 3).

### Results for the axis of scans

**Figure 4:**
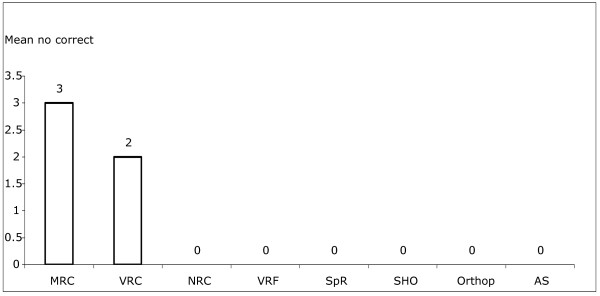
Mean scores for correct axis measurements out of 10 images for different grades of doctors.

One VRC attempted the axis of the scans. All answers were more than 90 degrees from the correct answer. All other participants chose to omit the axis of the scan in their answers. Mean scores were zero for all participants in the study (Figure 4).

## Discussion

As might be expected, non-medical subjects were very poor at OCT image interpretation, despite the fact that many of these staff are now trained to carry out the investigation. Moreover, this study has demonstrated that the level of seniority was positively correlated with the ability to correctly interpret OCT images, with increased competence identified with increasing seniority. Nevertheless, many senior doctors scored less than 50% of the total possible score. Consultants with a specialist retinal interest performed better than their non-retinal colleagues. All the junior doctors, regardless of seniority (SHO or SpR), scored similarly. The vast majority of doctors were unable to estimate either the correct average thickness of the lesion or the axis at which the scan was taken; whilst both axis and thickness are provided by the OCT software, the inability of subjects to estimate these parameters is a further indication of the lack of OCT knowledge and experience amongst clinicians.

All ophthalmologists would benefit from further training in the interpretation of OCT images, which could be readily incorporated into local teaching programmes. Currently, knowledge of OCT imaging is not included in the examination syllabus of the UK Royal College of Ophthalmologists. [6] Clearly there is a need to incorporate knowledge of this increasingly important imaging modality both in ophthalmological training and subsequent professional examinations.

In our opinion, OCT images should ideally be currently reported by consultants with a specialist interest in medical or surgical retina. When there is clinical diagnostic uncertainty relating to OCT interpretation by non-specialists or junior doctors, it is best that these images be reviewed by appropriately trained ophthalmologists prior to management decisions being taken.

## Conclusion

OCT imaging is an invaluable tool in the assessment of macular conditions, however, as this article highlights, all doctors would benefit from further training in OCT image interpretation. This could take place as formal teaching sessions or be included as part of an induction program.

## Authors' contributions

WHC conceived of the study, participated in its design, performed the statistical analysis and drafted the manuscript. JSS participated in the design of the study. MM participated in the design of the study and coordination. All authors read and approved the final manuscript.

## Pre-publication history

The pre-publication history for this paper can be accessed here:


